# Y-box binding protein-1 is crucial in acquired drug resistance development in metastatic clear-cell renal cell carcinoma

**DOI:** 10.1186/s13046-020-1527-y

**Published:** 2020-02-10

**Authors:** Ninadh M. D’Costa, Matthew R. Lowerison, Peter A. Raven, Zheng Tan, Morgan E. Roberts, Raunak Shrestha, Matthew W. Urban, Cesar U. Monjaras-Avila, Htoo Zarni Oo, Antonio Hurtado-Coll, Claudia Chavez-Munoz, Alan I. So

**Affiliations:** 1 Department of Urologic Sciences, Faculty of Medicine, University of British Columbia, Level 6, 2775-Laurel St, Vancouver, BC V5Z 1M9 Canada; 2 Vancouver Prostate Centre, 2660 Oak St., Vancouver, BC V6H 3Z6 Canada; 3 Department of Urology, Mayo Clinic College of Medicine and Science, Mayo Clinic, Rochester, MN USA; 4 Department of Radiology, Mayo Clinic, Rochester, MN USA

**Keywords:** Renal cell carcinoma, Angiogenesis, Metastasis, Sunitinib, Resistance, Tyrosine kinase inhibitors (TKI)

## Abstract

**Background:**

Renal cell carcinoma (RCC) is a highly vascular tumor and patients with low risk metastatic RCC of clear-cell histological sub-type (mccRCC) are treated with tyrosine-kinase inhibitors (TKIs), sunitinib, as the first-line of treatment. Unfortunately, TKI resistance eventually develops, and the underlying molecular mechanism is not well understood.

**Methods:**

RCC cell-line with metastatic clear-cell histology (Caki-1), and patient samples were analysed to identify the role of Y-box binding protein 1 (YB-1) and ATP-binding cassette sub-family B member 1 (ABCB-1) in acquired sunitinib-resistance development. Caki-1 was conditioned with increasing sunitinib doses to recapitulate acquired resistance development in clinics. Sunitinib-conditioned and wild-type Caki-1 were subjected to cell viability assay, scratch assay, chicken embryo chorioallantoic membrane engraftment and proteomics analysis. Classical biochemical assays like flow cytometry, immunofluorescent staining, immunohistochemical staining, optical coherence tomography imaging, Western Blot and RT-PCR assays were applied to determine the possible mechanism of sunitinib-resistance development and the effect of drug treatments. Publicly available data was also used to determine the role of YB-1 upregulation in ccRCC and the patients’ overall survival.

**Results:**

We demonstrate that YB-1 and ABCB-1 are upregulated in sunitinib-resistant in vitro, ex vivo, in vivo and patient samples compared to the sensitive samples. This provides evidence to a mechanism of acquired sunitinib-resistance development in mccRCC. Furthermore, our results establish that inhibiting ABCB-1 with elacridar, in addition to sunitinib, has a positive impact on reverting sunitinib-resistance development in in vitro*,* ex vivo and in vivo models.

**Conclusion:**

This work proposes a targeted therapy (elacridar and sunitinib) to re-sensitize sunitinib-resistant mccRCC and, possibly, slow disease progression.

## Background

Resistance to tyrosine kinase inhibitors (TKIs) is a concerning phenomenon for renal cell carcinoma (RCC) patients. RCC is among the top 10 cancers in the USA and 16th worldwide, being clear-cell RCC (ccRCC) the most prevalent histological subtype (> 80%) [1, 2]. Patients with localised tumor usually undergo partial or radical nephrectomy, but approximately 30% of the patients present with de novo metastatic disease (mRCC) [3]. RCC is a highly vascular tumor and sunitinib is the most commonly used anti-angiogenic targeted agent that acts by inhibiting receptor tyrosine kinases in endothelial cells. However, this study and other previously published research demonstrate the direct effect of sunitinib on cancer cells [4–6]. Current treatment decision for mRCC is purely based on the clinical features: low risk patients are usually treated with anti-angiogenic tyrosine kinase inhibitors (TKIs) and intermediate/severe risk patients with immunotherapy [7, 8]. However, initial responders to therapy will eventually develop resistance to TKIs within 10–14 months [3, 9]. The consequence of treatment failure in patients is deleterious due to the development of senescent phenotypes that contribute to tumor progression on therapy withdrawal [10]. Moreover, designing treatment strategies to overcome TKI resistance is challenging due to the lack of mechanistic insights and the availability of targeted therapies.

Y-box binding protein 1 (YB-1), a member of the cold-shock protein superfamily encoded by *YBX1* gene, is drastically increased in several types of cancer and it controls numerous cellular processes including DNA repair, transcription and translation of proteins [11–13]. Recently, it has been shown to have an association with pathogenic stages in RCC and metastasis [14, 15]. Furthermore, YB-1 has been involved in the “cross-talk” between mesangial and immune cells in inflammatory glomerular disease [16]. This could be a critical finding given immunotherapy’s role in the intermediate/severe risk mRCC patients [17–19].

On the other hand, ATP-binding cassette sub-family B member 1 (ABCB-1), plays a role in drug-resistance development in several cancers [20, 21]. This transporter has been shown to modulate cancer stem cell-like properties and epithelial–mesenchymal transition in non-small cell lung cancer [22]. In central nervous system, ABCB-1 upregulation restricts brain accumulation of dasatinib (TKI) limiting its effect in the patients [23].

Therefore, this study investigated the function of YB-1/ABCB-1 in acquired sunitinib-resistance development in mccRCC. Herein, we confirm the direct effect of sunitinib in cancer cells as well as demonstrate the association between YB-1 and ABCB-1 in sunitinib-resistance development in metastatic clear-cell RCC (mccRCC). We also propose a combination therapy to re-sensitize resistant mccRCC to sunitinib. Overall, this study reveals a possible mechanism of sunitinib-resistance development and a potential treatment strategy to improve survival in resistant mccRCC patients.

## Methods

### Cell culture and patient tissue samples

De-identified mccRCC tissue samples were obtained from patients after receiving informed consent in Vancouver General Hospital (H09–01628). Primary kidney tumor specimens from mccRCC patients with or without sunitinib treatment were considered for further analysis. Each group had more than 5 patient samples. Caki-1 (ATCC, VA, USA) was grown in McCoy’s 5A media (Gibco, MD, USA) supplemented with 10%FBS (Hyclone, UT, USA). 786-O (ATCC, VA, USA) was grown in RPMI media (Gibco, MD, USA) supplemented with 10%FBS (Hyclone, UT, USA). Human Umbilical Vein Endothelial Cells (HUVEC) from pooled donors (Lonza, GA, USA) were maintained in EBM-Plus Bulletkit (Lonza, GA, USA). Cells were passaged 0.25% Trypsin-EDTA (Gibco, MD, USA). Where appropriate, cell numbers were counted with Automated Cell Counter TC20 (Bio-Rad, WA, USA). All cells were incubated at 37 °C in 5% CO_2_.

### Reagents

The following reagents were purchased for this study: Sunitinib malate (Sutent, LC Laboratories, MA, USA); Elacridar (Toronto Research Chemicals, ON, CA); Mitomycin C and LY294002 (Sigma-Aldrich, MO, USA); AZD5363 and AZD8186 (Selleckchem, TX, USA); SL0101 (Calbiochem, CA, USA) and INK128 (Cayman Chemicals, MI, USA).

### Sunitinib-conditioned Caki-1 cell-line

Caki-1 DC cell-line was prepared from the parental Caki-1 as previously published [24]. Briefly, parental Caki-1 cells were grown to 50% confluence and then exposed to 0.1 μM sunitinib containing media. After 3–5 days, the media was replaced with fresh media for 24–48 h (Caki-1 DC, cycle1). Cells that showed proliferation were exposed to 25% higher concentration. The sunitinib on-off exposure cycle was maintained until approximately 20 cycles. In between each cycle, cells were allowed 5–8 passages. Caki-1 DC of cycle 15–18 were used for this study. Sunitinib-conditioned 786-ODC was also prepared from parental 786-O following the same procedure.

### Cell viability assay

Cells were seeded in 96-well plates at 4000 cells/well and incubated for 24 h. Different concentrations of drugs were added and media with DMSO ≤0.1% was used as control. After 72 h, treatment media was removed and MTS reagent (Sigma-Aldrich, MO, USA) in fresh media was added (1:20 ratio). The cells were then incubated at 37 °C, in 5% CO_2_, and plate readings were taken at 30 min and 1 h at 490 nm (BioTek, VT, USA). Each experiment had 3 technical replicates and the experiments were repeated at least 3 times.

### Scratch assay

Cells were allowed to grow to 80–90% confluence and, on the day of the experiment, were treated with 10 μg/ml of Mitomycin C for 2 h. The cells were scratched in a straight line with a sterile p200 tip, debris were removed by washes with phosphate-buffered saline (PBS; ThermoFisher Scientific, MA, USA), followed by incubation with appropriate cell medium. Images at time points and at matching reference coordinates were taken by an Axiovision microscope (Zeiss, ON, CA). Experiments were repeated at least 3 times.

### Silencing of YB-1

Knockdown of YB-1 in Caki-1WT/DC was carried out using esiYB-1 and non-specific esiEGFP was used as control (Sigma-Aldrich, MO, USA). The cells were transfected with RNAiMax transfecting reagent (ThermoFisher Scientific, MA, USA) by reverse transfection method. Briefly, a master mix of RNAiMAX reagent was prepared in OptiMEM media (ThermoFisher Scientific, MA, USA) at 4:1 siRNA to reagent ratio. Aliquots of only esiYB-1 and esiEGFP (SCR) were also prepared in OptiMEM media. The two preparations were gently mixed and incubated at room temperature. Meanwhile, cells were enzymatically detached, counted and reconstituted in OptiMEM media. Complexes were then gently added to the reconstituted cells and plated to a final concentration of 5 μM for esiYB-1 and SCR. After 48 h post-transfection, fresh OptiMEM media was added and after 72 h post-transfection, the cells were harvested. Experiments were repeated at least 3 times.

### Western blot

Western blots were carried out as previously published [25, 26]. Primary antibodies were incubated overnight at 4 °C: YB-1 (ENZO Life Sciences, NY, USA) at 1:1000 dilution, P-Glycoprotein (ABCB1) rabbit monoclonal (Abcam, MA, USA) at 1:500 dilution, P-Akt (S473), β-Catenin, GSK-3β, SOX2 and GAPDH (Cell Signalling, MA, USA) at 1:1000 dilution. Secondary antibodies were horseradish peroxidase (HRP) conjugated anti-mouse and anti-rabbit antibodies (Cell Signalling, MA, USA) for use with SuperSignal West Femto Maximum Sensitivity Substrate (ThermoFisher Scientific, MA, USA) and imaged using autoradiography films (Genesee Scientific, CA, USA). Band intensity was quantified using ImageJ software (NIH.gov). Experiments were repeated at least 3 times.

### Quantitative RT-PCR

RNA was extracted from cell lines using RNeasy Mini Kit (Qiagen Hilden, DE), according to the manufacturer’s instructions. Taqman-primers used for qPCR included YB-1, ABCB-1 and GAPDH (ThermoFisher Scientific, MA, USA). Amplification was performed using a Viia7 qPCR (Applied Biosystems, CA, USA). Target gene expression was normalized to GAPDH levels and the comparative cycle threshold (Ct) method was used to calculate relative quantification of target mRNAs. Each experiment had 3 technical replicates and the experiments were repeated at least 3 times.

### Immunofluorescence

Cells plated on coverslips (ThermoFisher Scientific, MA, USA) were allowed to grow for 48 h, fixed with 4% para-formaldehyde (Sigma-Aldrich, MO, USA), permeabilized with 0.1% Triton X-100 (Sigma-Aldrich, MO, USA) and blocked with 2.5% horse serum (Vector Laboratories, CA, USA). Coverslips were incubated overnight at 4 °C with anti-P-Glycoprotein (ABCB-1) mouse monoclonal antibody at 1:100 (Sigma-Aldrich, MO, USA) and anti-YB-1 rabbit monoclonal antibody at 1:500 dilution. Secondary antibody staining was performed with anti-rabbit Alexa-594 and anti-mouse Alexa-488 (Invitrogen, CA, USA), mounted with DAPI (Vector Laboratories, CA, USA) and imaged with confocal microscope at 20X and 60X magnifications (Olympus FV3000RS). Experiments were repeated at least 3 times.

### Immunohistochemistry

Formalin-fixed, paraffin-embedded tissue sections (4 μm) were deparaffinized by incubating slides at 60 °C for 1 h followed by repeated xylene and ethanol submersion. Antigen retrieval was performed with Diva Decloaker 10X (Cedarlane, ON, CA), steaming for 30 min, rinsed with dH_2_O and then incubated with 3% hydrogen peroxide (Sigma-aldrich, MO, USA). Sections were incubated with blocking from Vectastain ELITE ABC-Peroxidase kit according to manufacture’s protocol (Vector Laboratories, CA, USA). Slides were stained with rabbit monoclonal anti-ABCB-1 (1:100) and anti-YB-1 (1:500) antibodies overnight at 4 °C followed by secondary antibody staining using manufacturer’s protocol. Images were taken using SCN400 Slide Scanner (Leica Microsystems). Staining intensity was scored by a certified pathologist who was blinded to this study (score of 0–3). Cancer cells with positive staining in the tumor region were assigned an estimated percentage. Final intensity was calculated as: intensity = (score)×(percentage area)/100.

### Proteomics

Tumors from sunitinib-sensitive and resistant rodents were used to obtain difference in protein expression pattern using Tandem Mass Tag (TMT) labeling of peptides as published before [27]. For each group, tumor from 3 rodents were used.

### Flow cytometry

Cells were non-enzymatically detached from the plated with Cell Stripper (VWR, Cat# CA4500–668) and stained with Annexin-V (APC, BD Bioscience, Cat#550475) at 1:100 dilution for 1 h. The cells were stained for DAPI (BD Bioscience, NJ, USA) at 1:1000 dilution for 15 min and analysed by FACSCanto II Flow Cytometry System (BD Bioscience, NJ, USA). Percentage of positively stained cell count was quantified using FlowJo_V10. Each experiment had technical duplicates and the experiments were repeated at least 3 times.

### Engraftment of CAM tumor xenografts and imaging

Fertilized chicken eggs (Rudd, IA, USA) were incubated at 37 °C. On the fourth day of embryonic development (EDD-4), CAM assay was generated by transferring the egg contents into a plastic weight boat and incubated at 37 °C. On EDD-9, either Caki-1WT or Caki-1 DC cells were mixed with matrigel (BD Bioscience, NJ, USA) at 1 × 10^6^ cells/10 μL and pipetted into the CAM. On EDD-11, tumor pictures and measurements were taken (pre-treatment) and the tumor bearing embryos were randomly divided into each treatment groups. Topical drug dosage was administered daily until EDD-18 (endpoint). The optical imaging for each CAM at pre-treatment and endpoint were taken using a Nikon SMZ18 stereo-microscope at 4X magnification and were digitized using an integrated Nikon DS-Ri2 digital camera (Nikon, TYO, JP). Tumor volume was measured using optical coherence tomography. Each group had more than 3 CAM tumor bearing embryos and the experiment was repeated 2 times.

### Optical coherence tomography (OCT) imaging and analysis

OCT imaging was performed using a rapid 3D swept source Telesto 320C1 OCT system equipped with a telecentric scan lens (OCT-LK2) (Thorlabs Inc., NJ, USA). It has a center wavelength of 1300ηm, 3.0 μm axial resolution, 7.0 μm lateral resolution, a maximum imaging depth of 2 mm, and an A-line scan rate of 76 kHz. Imaging volumes of tumor bearing CAMs were acquired in a 5mmx5mmx2mm field of view at a resolution of 12μmx12μmx3.5 μm. Volumetric tumor image data.oct files were imported into MATLAB using code supplied by Thorlabs. Manual segmentation was performed on every tenth frame of OCT data using the MATLAB ‘imfreehand’ function to estimate for tumor volume. Changes in tumor volume were calculated by comparing the segmentation volumes from the pre-treatment and endpoint imaging datasets.

### Tumor xenografts

Animal studies were performed as published before [24] and in accordance to the guidelines of the Canadian Council on Animal Care with institutional certifications (University of British Columbia, A15–0231). Briefly, Caki-1WT/DC cells were injected subcutaneously (5 × 10^6^ cells) in the flank region of 8 weeks old nude mice (Charles Rivers Laboratories, MA, USA). Mice were randomly divided into groups after the tumors reached a volume of 100-200 mm^3^. Sunitinib malate was suspended in citrate-buffered solution (pH 3.5) and elacridar in diluent (0.5% methyl cellulose and 1% Tween-80 in ddH_2_O). Treatment was administered by oral gavage once daily for 5 days followed by 2 days off for 2–3 weeks. For the combination treatment, mice were treated with elacridar 15 min prior to the administration of sunitinib malate. Tumor volume was measured every 3 days using calipers and calculated: tumor volume (mm^3^) = length×width×height× 0.5. Each treatment group had more than 5 mice. Tumors were fixed with 10% para-formaldehyde (Sigma-Aldrich, MO, USA) for 24-48 h, 70% ethanol for 24 h (VWR International, PA, USA) followed by paraffin embedding.

### Statistical analysis

The data are represented by mean ± standard error of mean (SEM). Mean was used as ‘centre value’ where appropriate. Samples were normalized to the experimental control to a value of 1.0 or 100%, where appropriate. Difference between two groups were calculated using analysis of variance with Student’s *t*-test, two-sided. Multiple comparisons were calculated with ANOVA, corrected with Tukey’s test. Cell viability assays were analysed for IC_50_ using non-linear regression for normalised response-viability slopes. The trend in tumor volume was measured with linear regression analysis. All the graphs were prepared and analysed using GraphPad Prism 8 software. A *p* < 0.05 was considered statistically significant and differences were denoted by asterisks (*p < 0.05, **p < 0.01, ***p < 0.001 and ****p < 0.0001). All experiments were performed in triplicates and in three independent experiments.

### Code availability

The association of the gene expression with patients’ survival outcome was calculated using the median overall survival time from TCGA cBioPortal. Median as “NA” was used when the median value was not obtained at 50-percentile. The log-rank test in the “survival” R-package was used to generate the Kaplan-Meier plots. Death of a patient was used as the censored event in the survival analysis.

## Results

### Phenotypic variation between sunitinib-sensitive and resistant mccRCC samples

In order to investigate the mechanisms of sunitinib resistance, our laboratory has developed a sunitinib-conditioned cell-line (Caki-1 DC) by conditioning the parental mccRCC cells, (Caki-1WT) [26]. Moreover, it is widely accepted that ccRCC tumors commonly have Von Hippel-Lindau (VHL) gene mutation. However, our analysis from TCGA dataset shows that only ~ 50% of ccRCC patients have VHL mutation (Additional file 1: Figure S1). Therefore, we have conditioned both a VHL mutated 786-O (Additional file 1: Figure S1) and non-mutated Caki-1 ccRCC cell-lines.

**Fig. 1:**
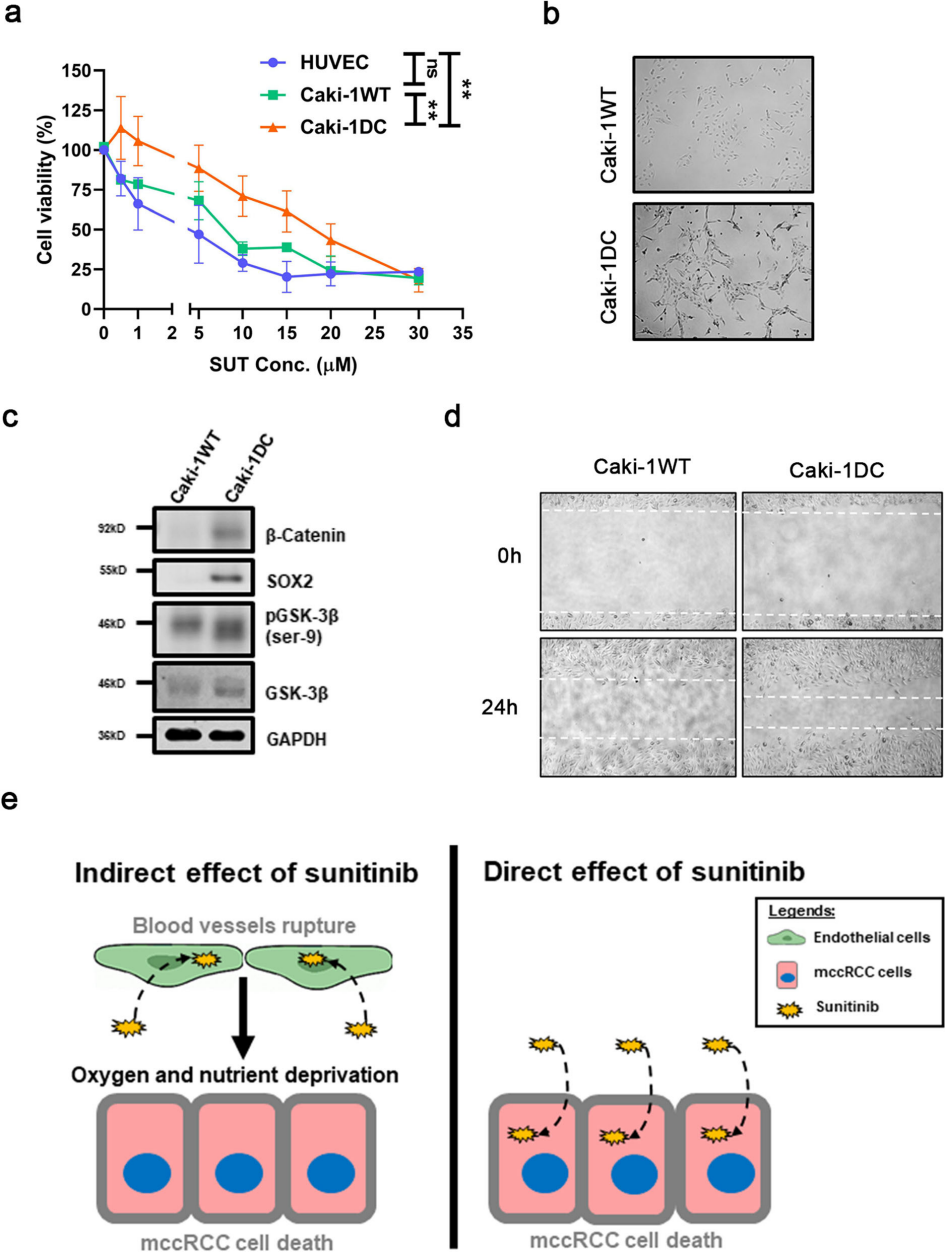
Phenotypic difference between sunitinib-resistant and sensitive mccRCC. **a** Endothelial cells (HUVEC), sunitinib-sensitive Caki-1WT and sunitinib-conditioned Caki-1 DC were exposed to different concentrations of sunitinib (SUT), and cell viability was measured by MTS assay (IC_50_ of HUVEC = 3.322 ± 0.558, Caki-1WT = 6.699 ± 0.781 and Caki-1 DC = 16.899 ± 1.383). **b** Phase contrast microscopy showing changes in cell morphology between Caki-1WT and Caki-1 DC. **c** Western blot showing increased protein levels of β-Catenin, SOX2 and GSK-3β that suggests cancer stem-cell like properties and epithelial-to-mesenchymal characteristics of Caki-1 DC vs. Caki-1WT. **d** Scratch assay showing increased migration of Caki-1 DC compared to Caki-1WT. **e** A schematic diagram showing the indirect and direct effects of sunitinib on cancer cells. Microscopic images were taken at 5X magnification. Data are mean ± SEM and normalised to matched controls. Results are representative of three independent experiments. *p < 0.05, **p < 0.01

Results from the cell viability curve demonstrated the tolerance to sunitinib by the Caki-1WT, Caki-1 DC and endothelial cells (HUVEC) (Fig. 1a). The endothelial cells and Caki-1WT were more sensitive to sunitinib compared to the conditioned Caki-1 DC (both *p* < 0.01) (Fig. 1a). Phase contrast microscopy revealed changes in cell morphology, showing a cobble-stone shape in Caki-1WT and spindle-like shape in Caki-1 DC (Fig. 1b). Similar morphological changes were also observed in another ccRCC cell-line, 786-O, that were conditioned to sunitinib (Additional file 1: Figure S1). We have also found increased β-catenin, SOX2 and GSK-3β protein expression between Caki-1 DC and Caki-1WT, which could suggest cancer stem-cell-like (CSC) and epithelial-mesenchymal transition (EMT) characteristics in Caki-1 DC (Fig. 1c) [28, 29]. Moreover, Caki-1 DC was found to migrate faster than Caki-1WT (Fig. 1d). Conventionally, sunitinib is known to affect angiogenesis by inhibiting endothelial cell proliferation, but some studies have shown direct effect of sunitinib on cancer cells (depicted in Fig. 1e) [23]. Our results show phenotypic differences between Caki-1WT and Caki-1 DC, suggesting a phenotypic switch once resistance develops.

### Direct effect of sunitinib on mccRCC cells

**Fig. 2:**
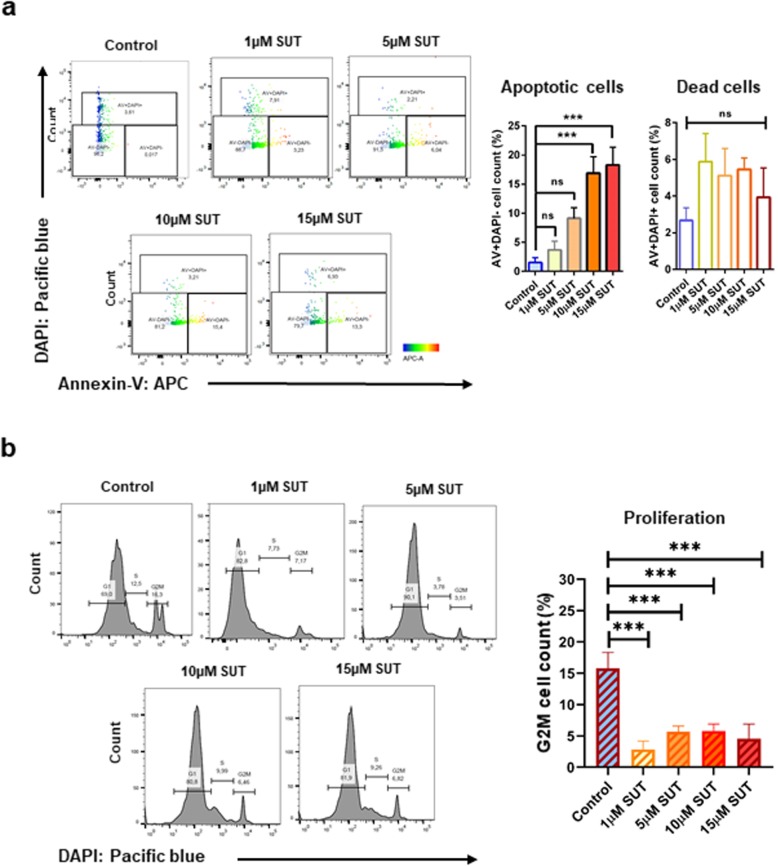
Direct effect of sunitinib on mccRCC cells. The parental mccRCC cell-line, Caki-1WT, was exposed to different concentrations of sunitinib (SUT) for 24 h. **a** Significant increase in apoptosis of the cells was observed with increasing concentration of the drug, but the population of non-apoptotic dead cells among different treatment groups were not significant. **b** With increasing concentrations of SUT, decrease in proliferation was observed with G2M phase using DAPI staining. Data are average of three independent experiments, mean ± SEM and normalised to matched controls. *p < 0.05, **p < 0.01, ***p < 0.001

To study the direct effect of sunitinib on mccRCC, we have treated Caki-1WT cells with different doses of sunitinib and stained with Annexin-V to analyse apoptotic cell death. We have observed significant increase in apoptotic cell population with 10 μM and 15 μM, but not with lower sunitinib doses (Fig. 2a, both *p* < 0.001). Moreover, the percentage of dead cells between low and high dose of sunitinib was not significant (Fig. 2a), suggesting that this direct effect is not due to cytotoxicity. Interestingly, the proliferation of Caki-1WT cells drastically decreased with only 1 μM of sunitinib (Fig. 2b, all *p* < 0.001). These results confirm that sunitinib has a direct effect on mccRCC cells leading to pronounced changes in apoptosis and proliferation of Caki-1WT.

### YB-1 and ABCB-1 increased expression in sunitinib-resistant mccRCC

**Fig. 3:**
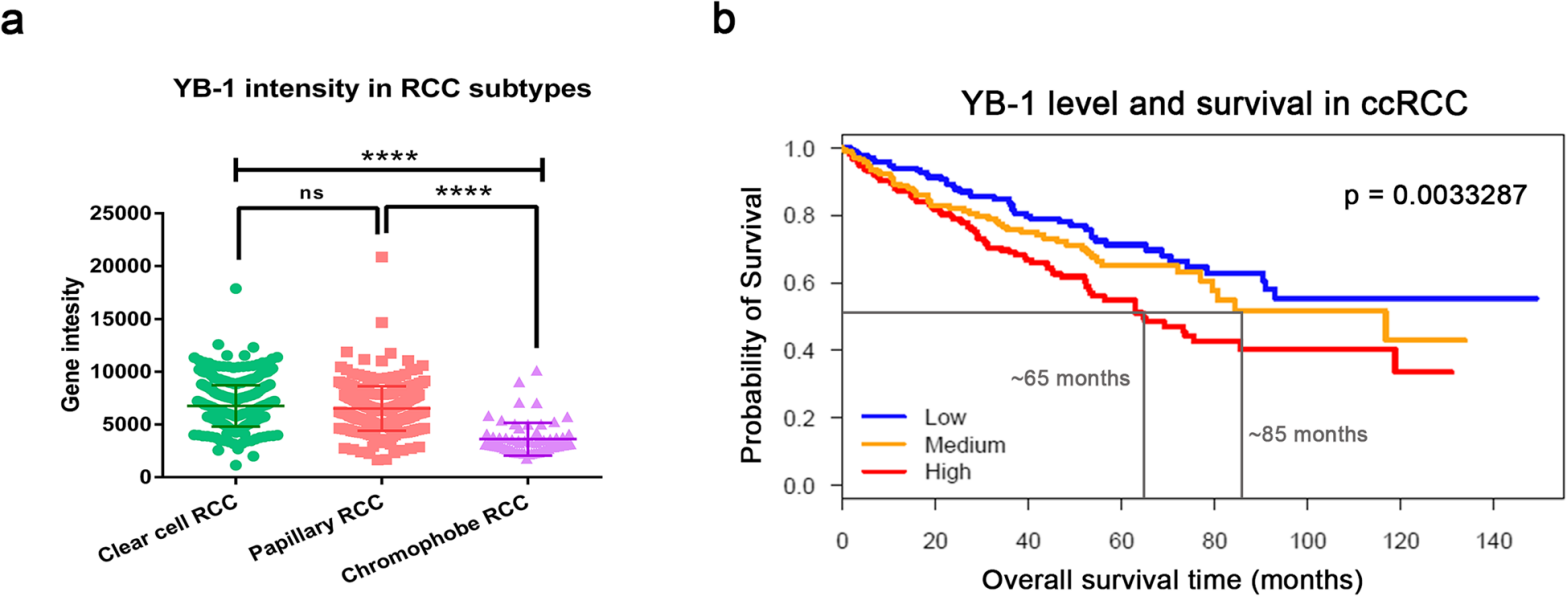
Expression levels of YB-1 and ABCB-1 in publicly available data. **a** A dot-plot on publicly available data showing high level of YB-1 gene expression intensity in ccRCC (449 patients) and pRCC (281 patients) compared to chRCC (65 patients). **b** A Kaplan-Meier curve showing significant lower probability of survival in patients with high YB-1 expression (median time of survival in High = 65 months, Medium = 85 months and Low = NA). Data are mean ± SEM (upper). *p < 0.05, **p < 0.01, ***p < 0.001, ****p < 0.0001

Besides establishing a sunitinib-conditioned cell-line, our laboratory has also established an acquired sunitinib-resistant mouse model [24]. Our proteomics analysis on tumors from the animal model showed an increased expression of ATP-binding cassette family of transporters (Additional file 1: Figure S2) [27]. Since YB-1 is upstream of many of these transporters, we analysed gene intensity of YB-1 in different sub-types of RCC. Results from The Cancer Genome Atlas (TCGA) Provisional dataset showed that YB-1 is highly upregulated in clear-cell and papillary subtypes compared to chromophobe subtype (Fig. 3a, both *p* < 0.001). Patients with clear-cell subtype were found to have decreased median time of survival with high YB-1 intensity (~ 65 months) compared to medium (~ 85 months) and low (NA) (Fig. 3b). To understand the prominence of YB-1 as a driver oncoprotein, we have also analysed the overall survival time in patients living with other cancer types from cBioPortal (Additional file 1: Figure S3). The Kaplan–Meier analysis showed that mutation in both *YBX1* and *ABCB1* genes lead to poor prognosis in patients compared to no alteration in those genes. Hence, we investigated the association of YB-1 and sunitinib-resistance development in mccRCC tumors.

**Fig. 4:**
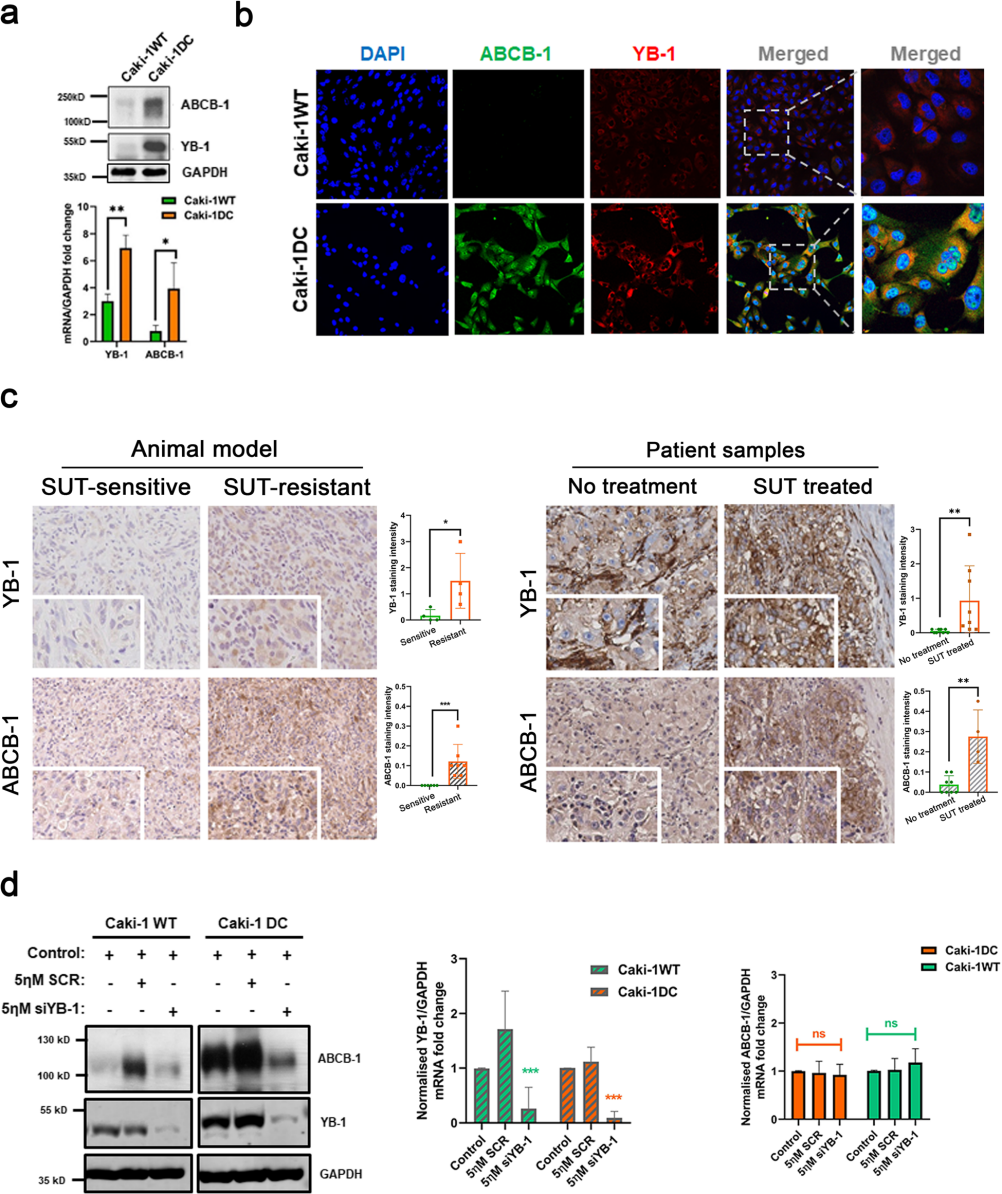
Increased expression of YB-1 and ABCB-1 in sunitinib-resistant compared to sunitinib-sensitive phenotypes. **a** Western blot and RT-PCR results show significant increase in YB-1 and ABCB-1 protein and mRNA levels in Caki-1 DC compared to Caki-1WT. **b** Increased YB-1 and ABCB-1 was also observed by immunofluorescence staining evaluation. **c** Immunohistochemical staining of YB-1 and ABCB-1 in our acquired sunitinib-resistant mouse model (*n* = 3–4) and patient samples (*n* = 5–7). **d** Western blot and RT-PCR results of YB-1 knockdown in Caki-1WT and Caki-1 DC showing significant downregulation of YB-1 protein and mRNA levels. The protein expression of its downstream target, ABCB-1, also decreased but the mRNA level remained unchanged. Data are mean ± SEM. Immunohistochemical images at scale bar 100 μm. Results are representative of three independent experiments. *p < 0.05, **p < 0.01, ***p < 0.0005, ****p < 0.0001

Our in vitro model showed upregulation and increased expression of YB-1 in Caki-1 DC when compared to Caki-1 WT (Fig. 4a-b). Immunohistochemical results from our in vivo model and patient samples, also showed significantly increased expression of both YB-1 and ABCB-1 protein levels (Fig. 4c). Therefore, we silenced YB-1 in Caki-1WT and Caki-1 DC using esiRNA, and obtained significant knockdown of YB-1 in both protein and mRNA levels (Fig. 4d). Moreover, knocking down YB-1 decreased ABCB-1 protein level. Similar results were observed with 786-O WT and DC cell-line (Additional file 1: Figure S1). The mRNA level of ABCB-1, however, did not change with esiYB-1 (Fig. 4d). For the first time, our data confirms the upregulation of YB-1 and ABCB-1 in acquired sunitinib-resistant mccRCC in vitro, in vivo models and patient samples. Furthermore, YB-1 dependent upregulation of ABCB-1, perhaps, leads to acquired sunitinib-resistance development in mccRCC tumors.

### Regulation of aberrant expression of YB-1/ABCB-1 in mccRCC

**Fig. 5:**
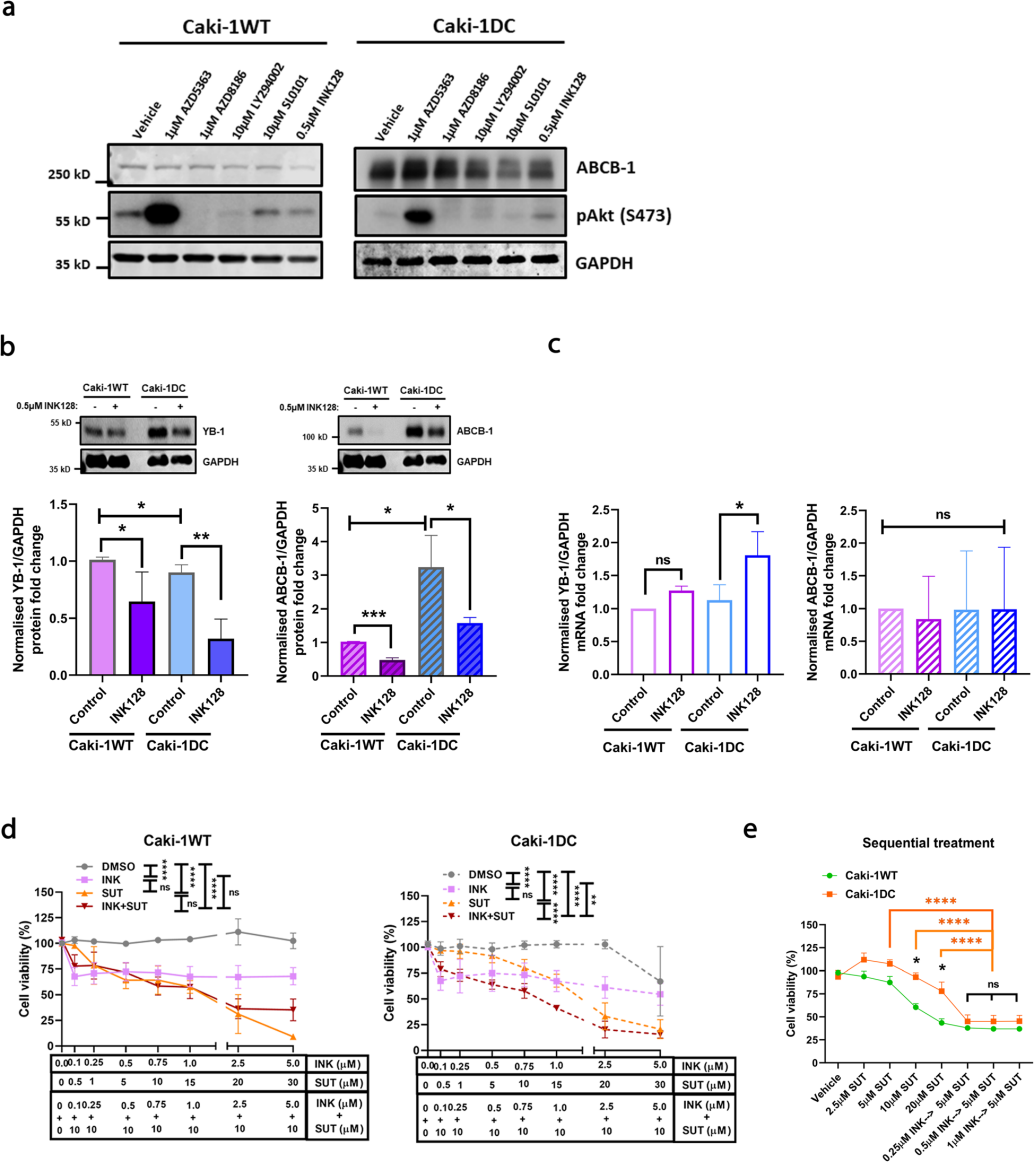
Inhibition of oncogenic pathways regulated aberrant expression of YB-1 and ABCB-1. **a** Different small-molecule inhibitors for Akt/PI3K, RSK and mTOR pathways show differential activation of Akt (phosphorylation at serine-473) and ABCB1 expression levels in Caki-1WT and Caki-1 DC. **b** Western blot results showing significant downregulation of YB-1 and ABCB-1 protein expression when treated with 0.5 μM INK128. **c** RT-PCR data shows a marked change in YB-1 mRNA level with 0.5 μM INK128 in Caki-1 DC compared to Caki-1WT, but no significant difference in ABCB-1 mRNA levels. **d** Cell viability assay demonstrating sensitization of Caki-1 DC cells to sunitinib. The response of Caki-1WT and Caki-1 DC are comparable, and a pronounced increase in cell death is observed with the combination therapy. **e** To simulate sequential treatment as applied in the clinic, Caki-1WT and Caki-1 DC were treated with different doses (0.25 μM, 0.5 μM and 1 μM) of INK128 for 48 h, washed off the drug with 1X PBS and then re-challenged with 5 μM SUT for 24 h to observe re-sensitization of Caki-1 DC to sunitinib. Our data shows significant cell death with sequential treatment and the drug-resistant phenotype Caki-1 DC had substantial effect, which is comparable to the parental Caki-1WT. SUT: sunitinib, AZD5363: Akt inhibitor, AZD8186: PI3K inhibitor, LY294002: Akt/PI3K pan inhibitor, SL0101: RSK inhibitor and INK128: mTOR inhibitor. Data are mean ± SEM and normalised to matched controls, *n* = 3–4 independent experiments. *p < 0.05, **p < 0.01, ***p < 0.0005, ****p < 0.0001

It is well known that protein kinase B (Akt), mammalian target of rapamycin (mTOR) and ribosomal S6 kinase (RSK) are upstream regulators of YB-1 [30–33]. Therefore, we tested different inhibitors against these oncogenic pathways (SUT: sunitinib, AZD5363: Akt inhibitor, AZD8186: Phosphoinositide 3-kinase (PI3K) inhibitor, LY294002: Akt/PI3K pan inhibitor, SL0101: RSK inhibitor and INK128: dual mTOR inhibitor) to determine their effect on YB-1 and ABCB-1 protein levels (Fig. 5a). A known potent mTOR inhibitor (0.5 μM INK128) showed reduction on YB-1 and ABCB-1 protein expression in Caki-1WT and Caki-1 DC cells (Fig. 5b). However, INK128 significantly upregulated YB-1 mRNA level in Caki-1 DC (~ 2.0 folds), which was not observed in Caki-1WT (Fig. 5c). Intriguingly, no significant difference was observed in ABCB-1 mRNA level after INK128 treatment (Fig. 5c).

To study the clinical relevance, we performed a cell viability assay and observed a significant decrease in cell viability in both Caki-1WT and Caki-1 DC after 72 h of INK128 treatment (Fig. 5d, *p* < 0.0001). However, this decrease did not change with increasing dose of the dual mTOR inhibitor, INK128. In Caki-1WT, no significant difference was observed in cell viability when treated with sunitinib monotherapy and sunitinib/INK128 combination treatment. Interestingly, the cell viability significantly decreased in Caki-1 DC when treated with a combination therapy of INK128 and 10 μM sunitinib compared to sunitinib monotherapy (Fig. 5d, *p* < 0.0001) (786-O cells, Additional file 1: Figure S1). To simulate a sequential treatment strategy as performed in the clinical practice, we treated our in vitro model with different concentrations of INK128 for 48 h (0.25 μM, 0.5 μM and 1 μM) followed by a low dose of sunitinib (5 μM) for 24 h, and then assayed for cell viability. There was a significant decrease in Caki-1 DC cell viability (~ 45%) in this sequential treatment compared to sunitinib monotherapy (~ 80%) (Fig. 5e, *p* < 0.001). These results suggest that downregulation of ABCB-1 in mTOR/YB-1 dependent pathway reverts sunitinib-resistance in mccRCC cells [34].

### Elacridar and sunitinib combination therapy in in vitro and ex vivo models

**Fig. 6:**
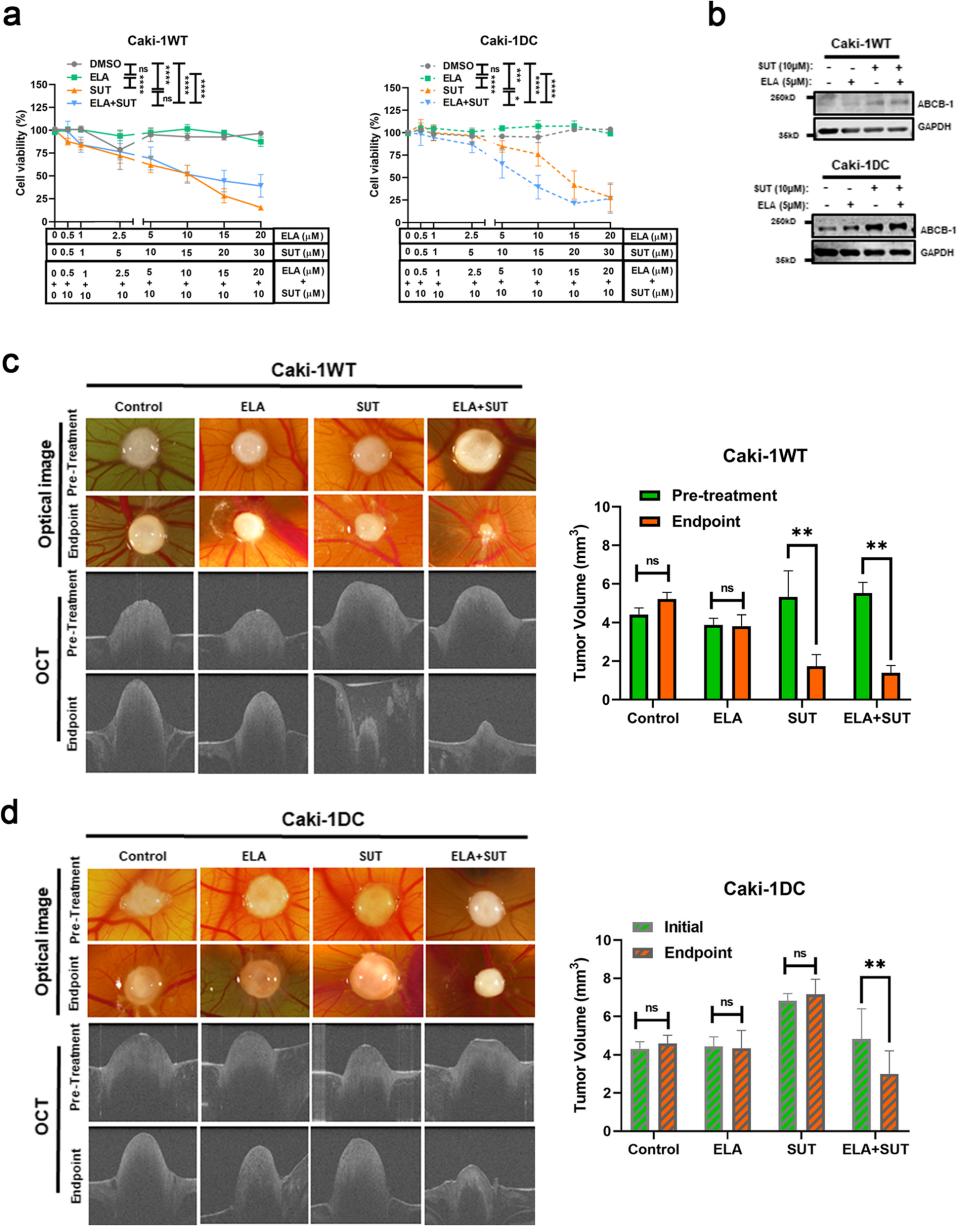
ABCB-1 inhibitor, elacridar, increases the efficacy of sunitinib. **a** Cell viability assay of sunitinib-sensitive Caki-1WT and the conditioned cell-line Caki-1 DC showing that ABCB-1 inhibition with elacridar significantly decreased cell viability of Caki-1 DC, which is comparable to Caki-1WT and **b**) Western blot showing slight increase of ABCB-1 protein level with sunitinib treatment in both Caki-1WT and Caki-1 DC, which did not change with elacridar. **c** Caki-1WT bearing embryos were treated with either vehicle, 10 μM SUT, 5 μM ELA or 10 μM SUT with 5 μM ELA combination treatment (left). The tumor size significantly decreased with SUT monotherapy and SUT with ELA combination treatment evaluated by optical image and optical coherence tomography (OCT) (bar-graph, right). **d** However, Caki-1 DC inoculated embryos responded only to 10 μM SUT and 5 μM ELA combination treatment and not to vehicle or monotherapies (bar-graph, right). SUT: sunitinib. ELA: elacridar. Data are mean ± SEM and normalised to matched controls, *n* = 3–5 independent experiments. Average of 3 to 5 CAM tumor bearing embryos. *p < 0.05, **p < 0.005, ***p < 0.0005, ****p < 0.0001

Previous reports from clinical trials show that resistance to mTOR inhibitors can also occur [35]. Therefore, we explored the possible use of ABCB-1 inhibitor, elacridar, to overcome acquired sunitinib-resistance in mccRCC. Cell viability assay showed that co-administration of 5 μM of elacridar with 10 μM of sunitinib significantly decreased cell survival in Caki-1 DC (~ 60%) compared to monotherapy (~ 90%) (Fig. 6a). With VHL-mutated 786-O WT and DC cells, similar results were obtained (Additional file 1: Figure S1). Interestingly, elacridar did not affect the protein level of ABCB-1, which slightly increased with sunitinib treatment, but still significantly decreased cell viability in both Caki-1WT and Caki-1 DC (Fig. 6b).

These observations were confirmed with Caki-1WT or Caki-1 DC engraftment in chicken embryo chorioallantoic membrane (CAM) ex vivo tumor model as well. The tumor bearing embryos were treated with either (DMSO ≤0.1%) vehicle as control group, 10 μM sunitinib, 5 μM elacridar or a combination of 10 μM sunitinib/ 5 μM elacridar for 7 days. For each embryo, tumor volume was measured before (pre-treatment) and after treatment (endpoint) by microscopy and optical coherence tomography (OCT). No significant difference was found between pre-treated and endpoint tumor size of Caki-1WT with vehicle or elacridar alone (Fig. 6c). However, a significant difference was observed when treated with sunitinib monotherapy (~ 2.5 folds) and sunitinib/elacridar combination treatment (~ 3.5 folds) (Fig. 6c, both *p* < 0.01). On the other hand, the Caki-1 DC tumors did not reduce in size when treated with vehicle, 5 μM elacridar or 10 μM sunitinib monotherapies (Fig. 6d), but significantly decreased only with the 5 μM elacridar/ 10 μM sunitinib combination treatment (Fig. 6d, *p* < 0.01).

### Sunitinib-resistant in vivo mccRCC tumors respond only to combination treatment

**Fig. 7:**
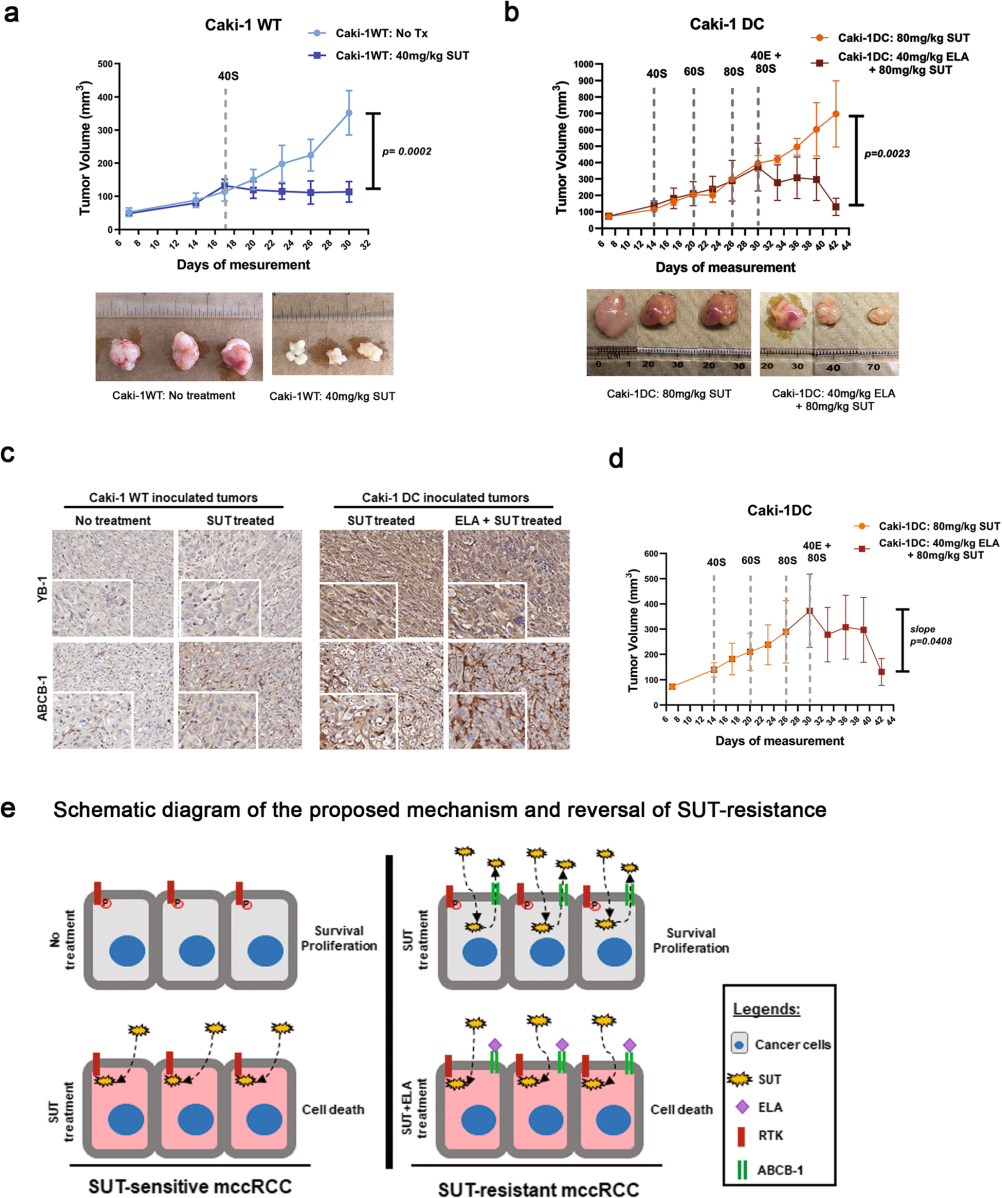
In vivo study using out sunitinib-resistant mccRCC mouse model. **a** Mice with Caki-1WT tumor responded to low dose of SUT (40 mg/kg, dark blue line) compared to vehicle treated mice (light blue line). **b** Caki-1 DC tumors kept growing while on SUT treatment and the dose escalated (40 mg/kg to 80 mg/kg, orange line). The tumor continued to grow in high dose of SUT therapy but the size decreased with the 80 mg/kg SUT with 40 mg/kg ELA combination therapy (red line). **c** Immunohistochemical staining of Caki-1WT and DC tumors for YB-1 and ABCB-1. **d** A graph comparing the rate of tumor growth (slope) within the same group of mice injected with Caki-1 DC that received combination therapy. The rate of tumor growth substantially decreased with the initiation of combination therapy compared to monotherapy in the same animals. **e** Schematic diagram of our proposed mechanism of sunitinib-resistance development and a, potential, therapy option to overcome sunitinib-resistance. SUT: sunitinib. ELA: elacridar. Data as mean ± SEM, *n* = 5–6 animals/group.*p < 0.05, **p < 0.005, ***p < 0.0005, ****p < 0.0001

In our in vivo model, Caki-1WT or Caki-1 DC tumors were inoculated in immunocompromised mice and were allowed to grow until the tumors reached 100mm^3^ in size [24]. We observed that Caki-1WT tumor size significantly decreased with 40 mg/kg sunitinib treatment (~ 5.0 folds) (Fig. 7a, *p* < 0.0001). In contrast, the Caki-1 DC tumors did not even respond to 80 mg/kg of sunitinib showing a drug-resistant phenotype. However, the combination of sunitinib (80 mg/kg) and elacridar (40 mg/kg) significantly decreased the tumor size (~ 3.5 folds) when compared to its matched tumor before and after therapy (Fig. 7b and d *p* < 0.01). Immunohistochemical staining of the Caki-1WT and DC inoculated tumors for YB-1 and ABCB-1 shows increased protein levels in Caki-1 DC compared to WT (Fig. 7c). Interestingly, the protein levels of both YB-1 and ABCB-1 did not change with combination therapy, which supports our in vitro results (Fig. 6b). Our result show that elacridar increased the efficacy of sunitinib in the resistant phenotype, which is depicted in the schematic diagram (Fig. 7e). This suggests that, in a clinical setting, oral co-administration of elacridar and sunitinib could be more therapeutically effective for sunitinib-resistant mccRCC patients.

## Discussion

Among all the four histological sub-types, ccRCC is the most vascular tumor and the effective use of anti-angiogenic drugs like sunitinib are essential in improving patient outcomes [36]. As sunitinib-resistance eventually develops in all treated TKI, it is imperative to gain insight into the molecular mechanism of acquired resistance to identify new treatments or methods to re-sensitize RCC. The effect of sunitinib on RCC is controversial with some data showing limited direct effect on RCC cells with anticancer effects due to inhibition of angiogenesis while other data suggests that sunitinib directly affects RCC cells [5, 6]. Possibly, this apparent contradiction is due to the different cell-lines used in these studies. These cell-lines were derived either from primary tumor or of dubious histology [37]. The current study, therefore, utilized Caki-1 cell-line, which is of metastatic origin with clear-cell RCC histology with wild-type VHL. We have also used another ccRCC cell-line, 786-O, that has VHL mutation.

We have established a sunitinib-conditioned in vitro*,* ex vivo and sunitinib-resistant in vivo model, which resembles resistant mccRCC characteristics in patients [24]. Our data show that endothelial cells (HUVEC) are sensitive to lower doses of sunitinib compared to Caki-1WT, but Caki-1 DC cells are tolerant to very high doses of sunitinib. Contrary to previous understanding, we show that sunitinib treatment significantly increases apoptosis and decreases proliferation in mccRCC cells providing evidence to a direct effect of sunitinib on cancer cells. To gain mechanistic insight into the differences between sunitinib-sensitive and resistant phenotypes, proteomics analysis on our in vivo model was performed. Our results indicate the involvement of ATP-binding cassette transporters, which are pivotal in drug resistance development in many cancers [16, 38]. As YB-1 is an upstream regulator of many of these transporters, we have leveraged patient data from TCGA (cBioPortal). The analysis showed a marked decrease in median overall survival time in ccRCC patients with high YB-1 gene intensity compare to medium and low gene intensities. Moreover, in other cancer types, patients with mutations of YB-1 and ABCB-1 genes have decreased overall survival time. Recently, a study established the importance of YB-1 in mccRCC cell migration and adhesion by activation of nuclear factor kappa B (NF-κB) signalling pathway [39]. Therefore, it is a logical extension to investigate the association of YB-1/ABCB-1 and sunitinib-resistance development in mccRCC tumors.

In our in vitro and in vivo models, we observed a significant increase in YB-1 and ABCB-1 protein and mRNA levels in the sunitinib-resistant samples compared to the sensitive samples. Moreover, knocking down YB-1 substantially downregulates ABCB-1 protein levels. This mechanistic insight is important because YB-1/ABCB-1 pathway is involved in survival, immune response, relapse and distant metastasis in patients. As a result, we tested numerous inhibitors against the common oncogenic pathways that are known to regulate YB-1 and ABCB-1 expression. However, drastic difference in ABCB-1 protein expression was only observed with very low dose of the dual mTOR inhibitor (0.5 μM INK128). Furthermore, a previously published study showed that YB-1 expression is regulated by mTOR pathway [33]. This is intriguing because, once resistance develops, mTOR inhibitors are considered an option as the second-line of treatment for mccRCC patients [34]. We show that the dual mTOR inhibitor (INK128) significantly reduced both YB-1 and ABCB-1 protein levels.

When treated with INK128, the change in mRNA level of YB-1 in Caki-1WT is not significant but is highly significant in Caki-1 DC. This could be explained by the effect of mTOR pathway in the translation of proteins and that inhibition of this pathway leads to accumulation of mRNA. Interestingly, the mRNA level of ABCB-1 remained non-significant in both Caki-1WT and Caki-1 DC. We have also observed no significant change in ABCB-1 mRNA level with siYB-1. These results suggest that mTOR pathway affects the translation of YB-1 protein but not of ABCB-1. It could be speculated that downregulation of YB-1 with either siYB-1 or mTOR inhibitor leads to increased ABCB-1 protein degradation, which is why a marked decrease in ABCB-1 protein level is observed but not in mRNA level. This mechanism of action of mTOR inhibitor could partially explain the success behind RECORD-3 clinical trial where a sequential treatment of sunitinib followed by everolimus had improved overall survival in patients [40]. This sequential treatment strategy was simulated in our laboratory by re-challenging the sunitinib-conditioned Caki-1 DC cells with sunitinib after the dual mTOR inhibitor treatment and observed sensitization of Caki-1 DC using lower doses of sunitinib. For the first time, our study provides a possible mechanistic insight into the rationale use of mTOR inhibitors as a second-line of therapy in sunitinib-resistant mccRCC patients.

Unfortunately, it is well known that a combination of mTOR inhibitor and sunitinib is highly toxic in clinical trials, therefore, is not a clinically feasible option [41]. On the other hand, an ABCB-1 blocker, elacridar, increases treatment efficacy in glioblastoma patients by overcoming blood-brain barrier [42]. Moreover, a study on lysosomal sequestration of sunitinib in RCC, proposed the use of elacridar to increase the efficacy of sunitinib. However, the study did not elucidate the mechanism of increased ABCB-1 expression in sunitinib treated cells and used only in vitro model of 786-O cell line, which is of primary ccRCC origin [43]. Therefore, the current study investigated the use of ABCB-1 inhibitor, elacridar, in mccRCC. Our data provides evidence that co-administration of sunitinib and elacridar significantly reduced cell viability in Caki-1 DC compared to sunitinib alone. To further support our hypothesis, we have generated chicken embryo chorioallantoic membrane (CAM) ex vivo tumor model with Caki-1WT and Caki-1 DC cells. In this assay, tumors engrafted with Caki-1WT (sunitinib-sensitive) significantly decreased in size when treated with either sunitinib alone or sunitinib/elacridar combination therapy. On the other hand, Caki-1 DC tumors (sunitinib-resistant), responded only to sunitinib/elacridar combination therapy. This observation was also confirmed in our in vivo model. Oral administration of 40 mg/kg of sunitinib significantly reduced the tumor size in Caki-1WT inoculated mice but had no effect on Caki-1 DC tumors. The Caki-1 DC tumors continued to grow despite increasing the dose of sunitinib (40 mg/kg to 80 mg/kg). However, the Caki-1 DC tumor size significantly reduced in the 40 mg/kg elacridar and 80 mg/kg sunitinib group treated compared to the 80 mg/kg sunitinib monotherapy treated group. This reduction in Caki-1 DC tumor size is also significant when compared to its matched pre-treated tumors. One limitation of our study is the use of immunocompromised models. As a result, the effect of YB-1/ABCB-1 pathway in pro-migratory immune cell mediated inflammation was not investigated. Hypothetically, the use of these inhibitors may also help in modulating tumor microenvironment in mccRCC tumors that could dictate response to immunotherapies.

## Conclusions

Overall, the current study demonstrates (i) the direct effect of sunitinib on mccRCC cells, (ii) this direct effect leads to several phenotypic changes in mccRCC, (iii) chronic sunitinib treatment develops acquired drug-resistance partly through YB-1/ABCB-1 mediated pathway and (iv) blocking ABCB-1 with elacridar has shown to overcome sunitinib-resistance in mccRCC samples. The results from this study provide mechanistic insight into the dynamic nature of mccRCC tumors following sunitinib therapy and proposes a potential treatment option to overcome the deleterious effects of resistance development in advanced kidney cancer patients.

## Supplementary information

**Additional file 1.** Supportive data for manuscript that are not shown in the main figures are provided as supplementary materials in Additional file 1: Figures 1, 2 and 3.

## Data Availability

The datasets from the current study are available from the corresponding author on reasonable request. Publicly available data was obtained from the cBioPortal (https://www.cbioportal.org/).

## Abbreviations

ABCB-1: ATP-binding cassette sub-family B member 1
Akt: Protein kinase B
Caki-1 DC: Caki-1 sunitinib drug-conditioned
Caki-1WT: Caki-1 wild-type
CAM: Chicken embryo chorioallantoic membrane
ccRCC: Clear-cell renal cell carcinoma
CO_2_: Carbon dioxide
EDD: Embryonic development day
ELA: Elacridar
esiRNA: Endoribonuclease-prepared small interfering RNA
GAPDH: Glyceraldehyde 3-phosphate dehydrogenase
HUVEC: Human umbilical cord endothelial cells
IHC: Immunohistochemistry
mccRCC: Metastatic clear-cell renal cell carcinoma
mTOR: Mammalian target of rapamycin
NF-κB: Nuclear Factor kappa B
OCT: Optical coherence tomography
RCC: Renal cell carcinoma
RSK: Ribosomal S6 kinase
RT-PCR: Real time polymerase chain reaction
SUT: Sunitinib
TKI: Receptor tyrosine kinase inhibitors
YBX1: Y-box binding protein 1
