# Supplementary material for: Y-box binding protein-1 is crucial in acquired drug resistance development in metastatic clear-cell renal cell carcinoma
Source: J Exp Clin Cancer Res. 2020 Feb 10;39:33. doi: 10.1186/s13046-020-1527-y (PMC7011538; doi:10.1186/s13046-020-1527-y)
Supplement: Supplementary file 1 — Additional file 1. Supportive data for manuscript that are not shown in the main figures are provided as supplementary materials in Additional file 1: Figures 1, 2 and 3. [file 13046_2020_1527_MOESM1_ESM.pdf]

Additional File

Y-box binding protein-1 is crucial in acquired drug resistance development in metastatic clear-cell renal cell carcinoma

Authors: Ninadh M. D'Costa[1,4], Matthew R. Lowerison[2], Peter A. Raven[1], Zheng Tan[1], Morgan E. Roberts[1,4], Raunak Shrestha[1], Matthew W. Urban[3], Cesar U. Monjaras-Avila[1,4], Htoo Zarni Oo[1,4], Antonio Hurtado-Coll[1,4], Claudia Chavez-Munoz[1,4] and *Alan I. So[1,4].

Affiliations:

[1]Department of Urologic Sciences, Faculty of Medicine, University of British Columbia, Vancouver BC, Canada

[2]Department of Urology, Mayo Clinic College of Medicine and Science, Mayo Clinic, Rochester, MN, USA.

[3]Department of Radiology, Mayo Clinic, Rochester, MN, USA

[4]Vancouver Prostate Centre, 2660 Oak St. Vancouver BC V6H 3Z6, Canada.

Running title: YB-1/ABCB-1 in sunitinib-resistant kidney tumors

***Corresponding author:**
Dr. Alan I. So,
Department of Urologic Sciences, University of British Columbia,
Level 6, 2775-Laurel St, Vancouver, BC, Canada, V5Z 1M9,
Email: dralanso@mail.ubc.ca
Tel: +1- 604-875-5603
Fax: +1- 604-875-5604

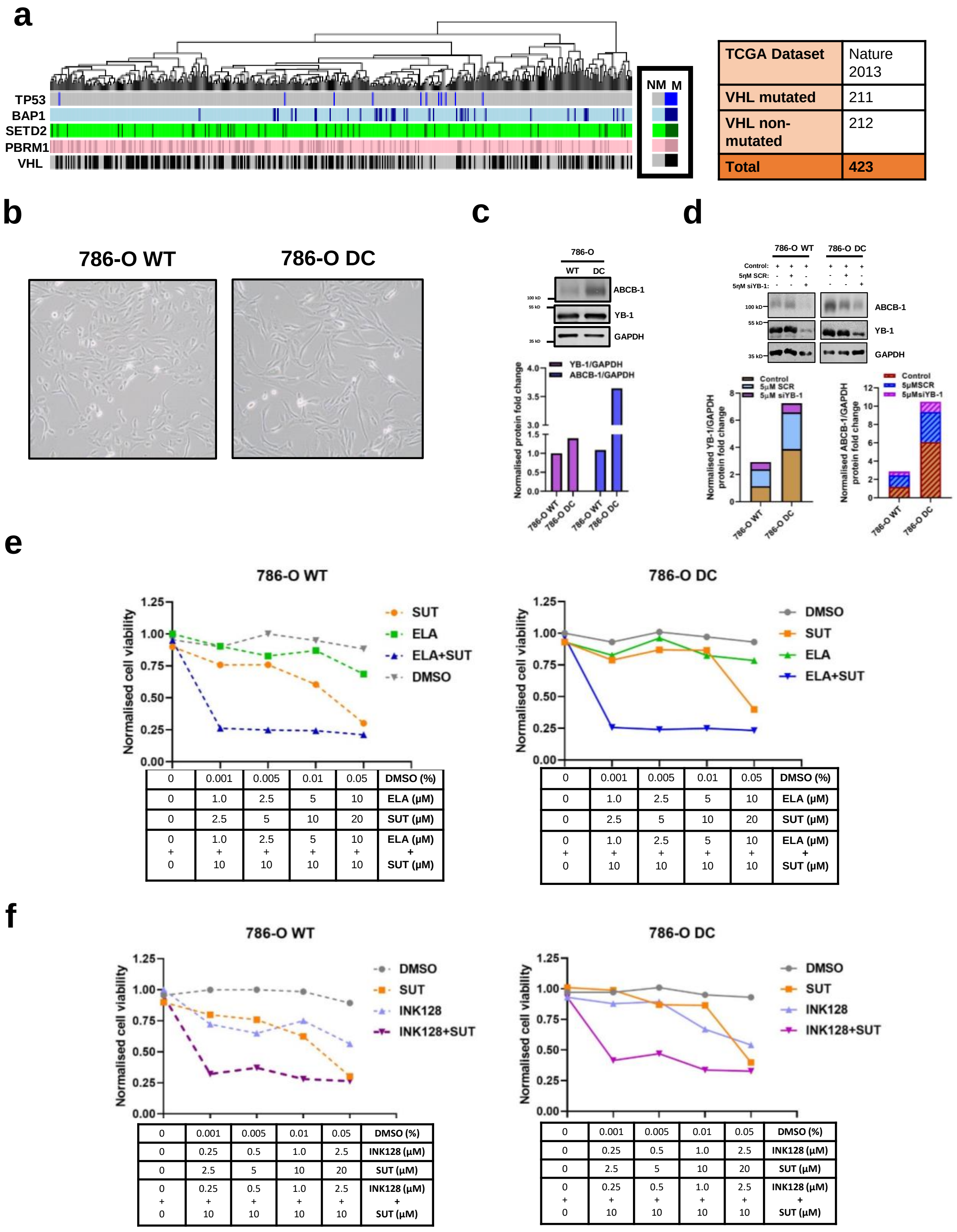
a
TP53
BAP1
SETD2
PBRM1
VHL
NM M
TCGA Dataset | Nature 2013
VHL mutated | 211
VHL non-mutated | 212
Total | 423
b
786-O WT
786-O DC
c
786-O
WT DC
ABCB-1
YB-1
GAPDH
YB-1/GAPDH
ABCB-1/GAPDH
Normalised protein fold change
786-O WT
786-O DC
d
786-O WT
786-O DC
Control:
5ηM SCR:
5ηM siYB-1:
ABCB-1
YB-1
GAPDH
Control
5μM SCR
5μM siYB-1
Normalised YB-1/GAPDH protein fold change
Control
5μMSCR
5μMsiYB-1
Normalised ABCB-1/GAPDH protein fold change
e
786-O WT
SUT
ELA
ELA+SUT
DMSO
Normalised cell viability
0 | 0.001 | 0.005 | 0.01 | 0.05 | DMSO (%)
0 | 1.0 | 2.5 | 5 | 10 | ELA (μM)
0 | 2.5 | 5 | 10 | 20 | SUT (μM)
0 + 0 | 1.0 + 10 | 2.5 + 10 | 5 + 10 | 10 + 10 | ELA (μM) + SUT (μM)
786-O DC
DMSO
SUT
ELA
ELA+SUT
Normalised cell viability
0 | 0.001 | 0.005 | 0.01 | 0.05 | DMSO (%)
0 | 1.0 | 2.5 | 5 | 10 | ELA (μM)
0 | 2.5 | 5 | 10 | 20 | SUT (μM)
0 + 0 | 1.0 + 10 | 2.5 + 10 | 5 + 10 | 10 + 10 | ELA (μM) + SUT (μM)
f
786-O WT
DMSO
SUT
INK128
INK128+SUT
Normalised cell viability
0 | 0.001 | 0.005 | 0.01 | 0.05 | DMSO (%)
0 | 0.25 | 0.5 | 1.0 | 2.5 | INK128 (μM)
0 | 2.5 | 5 | 10 | 20 | SUT (μM)
0 + 0 | 0.25 + 10 | 0.5 + 10 | 1.0 + 10 | 2.5 + 10 | INK128 (μM) + SUT (μM)
786-O DC
DMSO
SUT
INK128
INK128+SUT
Normalised cell viability
0 | 0.001 | 0.005 | 0.01 | 0.05 | DMSO (%)
0 | 0.25 | 0.5 | 1.0 | 2.5 | INK128 (μM)
0 | 2.5 | 5 | 10 | 20 | SUT (μM)
0 + 0 | 0.25 + 10 | 0.5 + 10 | 1.0 + 10 | 2.5 + 10 | INK128 (μM) + SUT (μM)

Figure 1

Additional File. Figure 1: VHL mutated cell-line 786-O also develop acquired sunitinib-resistance by YB-1/ABCB-1 pathway. a) Gene mutation data from TCGA KIRC dataset showing 212 ccRCC patients are VHL mutated and 211 non-mutated (total ccRCC patient = 423, shown in the table), b) morphological changes observed in 786-O WT and DC, c) YB-1 and ABCB-1 protein levels in 786-O WT and DC, d) knocking down YB-1 with siYB-1 also decreased ABCB-1 protein level, e) treatment response of 786-O WT and DC to ABCB-1 blocker, elacridar, with/without sunitinib, and f) with mTOR inhibitor, INK128. SUT: sunitinib, ELA: elacridar, SCR: esiEGFP, siYB-1: esiYB1.

Gene	Accession#	Name	Peptide#	Log FC_80 mg/kg	t	Score	p-value	FDR
ABCB1	P08183	Multidrug resistance protein-1	26	-0.45323	-2.29642	0.053957	4.10E-11	9.55E-09
ABCB10	Q9NRK6	ATP-bonding cassette sub-family B member 10	5	0.267708	1.8302	0.108369	0.010748	0.152629
ABCC4	O15439	Multidrug resistance-associated protein 4	12	0.473752	2.73992	0.027898	6.12E-08	7.98E-06
ABCD1	P33897	ATP-bonding cassette sub-family D member 1	6	-0.30498	-1.41874	0.197421	0.042111	0.34299
ABCD3	P28288	ATP-bonding cassette sub-family D member 3	12	-0.22344	-1.26589	0.244637	0.02586	0.262226
ABCE1	P61221	ATP-bonding cassette sub-family E member 1	16	0.195736	1.60078	0.151901	0.000465	0.016489
ABCF2	Q9UG63	ATP-bonding cassette sub-family F member 2	20	0.23689	1.651535	0.14105	5.74E-05	0.003047

Additional File. Figure 2: Proteome data analysis. Tumors from sunitinb-sensitive and resistant rodents were analysed for difference in protein expression pattern. Significant upregulation of ATP-binding cassette transporter proteins were observed in sunitinib-resistant tumors compared to the sensitive tumors. ABCB-1 was the most markedly upregulated among other transporters from the same family of proteins. Individual p-values were calculated using Student’s t-test, n=3 animals/group.

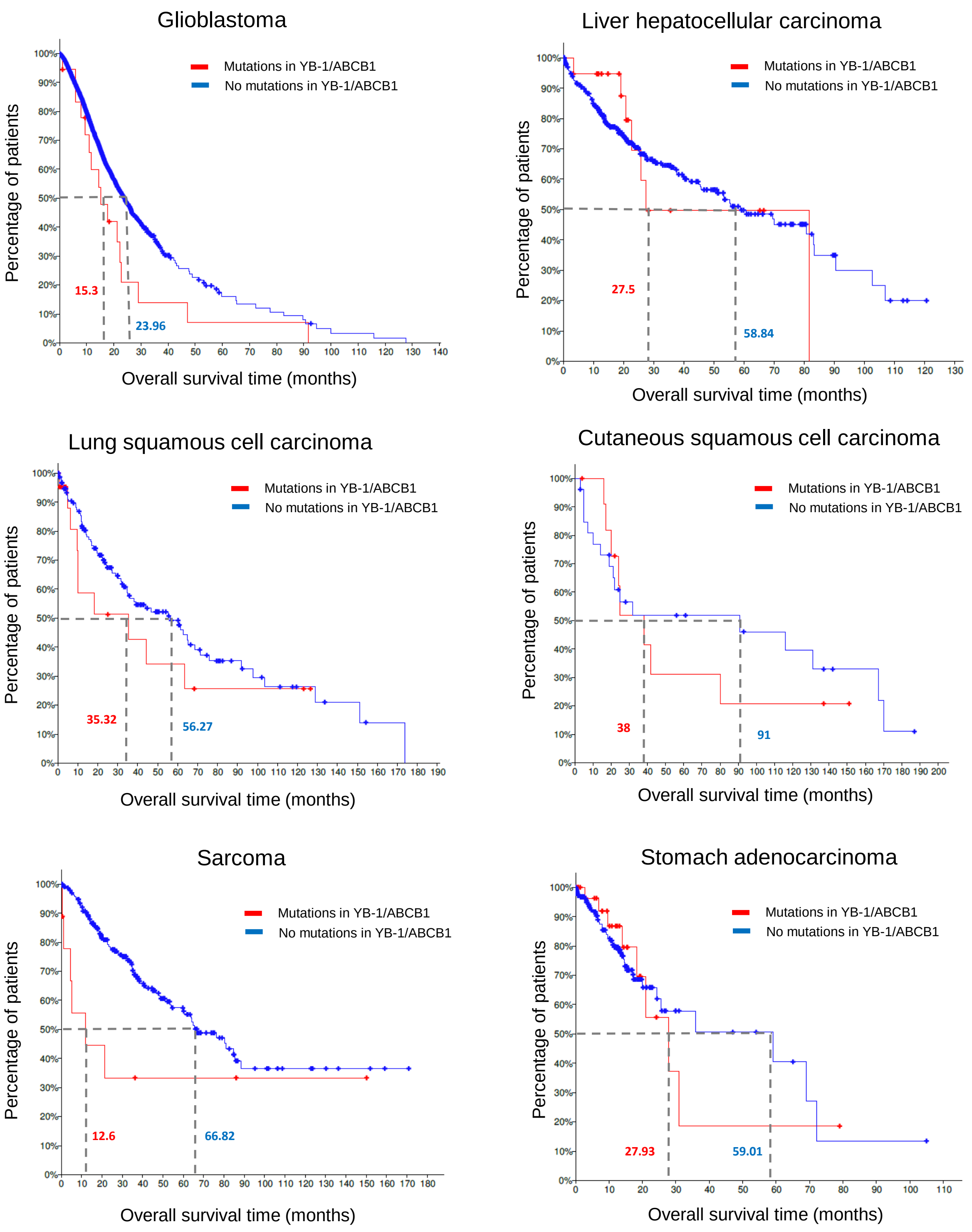
Glioblastoma
Mutations in YB-1/ABCB1
No mutations in YB-1/ABCB1
Percentage of patients
Overall survival time (months)
15.3
23.96
Liver hepatocellular carcinoma
Mutations in YB-1/ABCB1
No mutations in YB-1/ABCB1
Percentage of patients
Overall survival time (months)
27.5
58.84
Lung squamous cell carcinoma
Mutations in YB-1/ABCB1
No mutations in YB-1/ABCB1
Percentage of patients
Overall survival time (months)
35.32
56.27
Cutaneous squamous cell carcinoma
Mutations in YB-1/ABCB1
No mutations in YB-1/ABCB1
Percentage of patients
Overall survival time (months)
38
91
Sarcoma
Mutations in YB-1/ABCB1
No mutations in YB-1/ABCB1
Percentage of patients
Overall survival time (months)
12.6
66.82
Stomach adenocarcinoma
Mutations in YB-1/ABCB1
No mutations in YB-1/ABCB1
Percentage of patients
Overall survival time (months)
27.93
59.01

Figure 3

Additional File. Figure 3: Mutation of YBX1 and ABCB1 gene in other types of cancer and the overall survival in patients. To investigate the relevance of YBX1/ABCB1 pathway in other cancer types, we analyzed patient data from TCGA cBioPortal for overall survival in patients. As shown in the Kaplan-Meier curves, drastic decrease in survival time is observed in glioblastoma, hepatocellular carcinoma, lung squamous cell carcinoma, cutaneous squamous cell carcinoma, sarcoma and stomach adenocarcinoma patients when tumors had mutation in YBX1 and ABCB1 genes (red) compared to the tumors with no mutation (blue).
